# Dermatofibrosarcoma protuberans in a young patient with epidermolysis bullosa: a case report

**DOI:** 10.1186/s12893-021-01105-6

**Published:** 2021-02-23

**Authors:** B. Bonaventura, D. Kraus, G. B. Stark, H. Fuellgraf, J. Kiefer

**Affiliations:** 1 Department of Plastic and Hand Surgery, Medical Center - University of Freiburg, Faculty of Medicine, Comprehensive Cancer Center Freiburg, University of Freiburg, Freiburg, Germany; 2 Institute for Surgical Pathology, Medical Center, University of Freiburg, Freiburg, Germany

**Keywords:** Dermatofibrosarcoma protuberans, Epidermolysis bullosa, Local skin flap, Reconstructive surgery, Case report

## Abstract

**Background:**

Epidermolysis bullosa is a group of rare inherited skin diseases characterized by blister formation following mechanical skin trauma. Epidermolysis bullosa is associated with increased skin cancer rates, predominantly squamous cell carcinomas, yet to our best knowledge, there is no reported case of dermatofibrosarcoma protuberans in a patient with Epidermolysis bullosa.

**Case presentation:**

Here, we present a 26-year-old man with junctional epidermolysis bullosa, who developed a DFSP on the neck. Initial, the skin alteration was mistakenly not considered malignant, which resulted in inadequate safety margins. The complete resection required a local flap to close the defect, which is not unproblematic because of the chronic inflammation and impaired healing potential of the skin due to Epidermolysis bullosa.

**Conclusions:**

To our best knowledge, this is the first reported case of a skin-associated sarcoma in a patient with EB; however, further investigation is required to verify a correlation.

## Background

Epidermolysis bullosa (EB) is a rare inheritable genetic skin disease presented in four different forms with multiple subtypes. The United States’ overall incidence is approximately 19 per million live births, with a prevalence of 8 per million population, while the worldwide incidence is estimated at 1 per 100,000 live births [1]. Patients suffer from skin fragility with blisters following minor trauma, which further results in chronic skin wounds with permanent inflammation and impaired wound healing [2, 3]. The severity of symptoms differs from mild cases to lethality in the first two years of life, depending on the underlying genetic mutation. Patients with junctional epidermolysis bullosa usually present with generalized skin fragility, but mild expressions are possible. Skin tumors, most notably squamous cell carcinoma (SCC), are linked to EB. While SCC has been found to have a higher prevalence in all types of EB, patients with the recessive dystrophic form of EB predominantly develop it [4, 5]. Dermatofibrosarcoma protuberans (DFSP) is a sporadic skin tumor but the most common of all skin sarcomas (less than 1 case per 100,000 population) [6]. Most commonly, DFSP occurs at the trunk or proximal extremities as a relatively inconspicuous, slowly growing plaque with red or brownish discoloration [7, 8]. Atypical locations, e.g., the distal extremities or acres, are not related to higher mortality [9]. The specific characteristics of DFSP, such as asymmetric horizontal growth and infiltration of deeper structures, differ from other skin tumors [10, 11]. Metastases are rare and occur in less than 5% of the patients [12]. Surgical removal of the tumor with wide safety margins is crucial as minor safety margins are associated with higher rates of local DFSP recurrence [7, 12, 13].

To our best knowledge, there is no reported case of skin-associated sarcoma, specifically dermatofibrosarcoma protuberans, in patients with epidermolysis bullosa.

## Case presentation

Here, we present a 26-year-old male with junctional epidermolysis bullosa (generalized, intermediate form) who suffered from a skin lump on his neck, which surprisingly turned out to be a dermatofibrosarcoma protuberans. Informed consent was obtained from the patient prior to data acquisition.

**Fig. 1:**
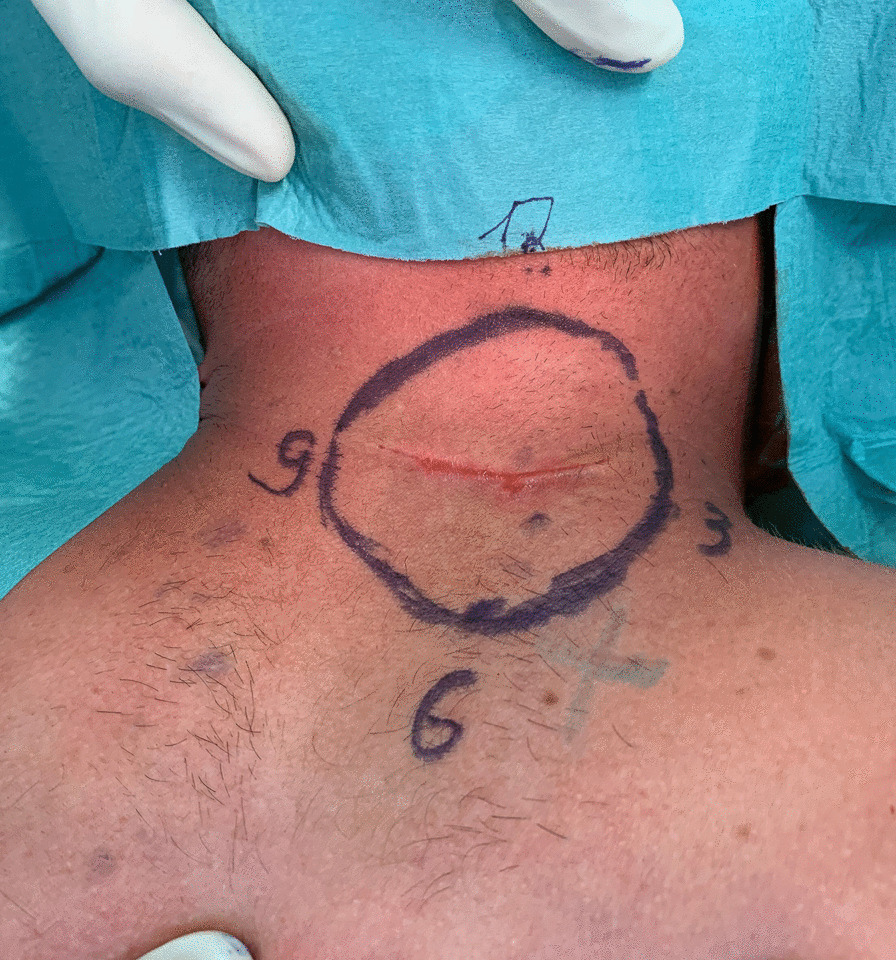
Preoperative marking of the estimated skin resection ensures adequate safety margins, including the scar from the initial biopsy (incomplete resection). Orientation markings are essential for a complete histological workup

**Fig. 2:**
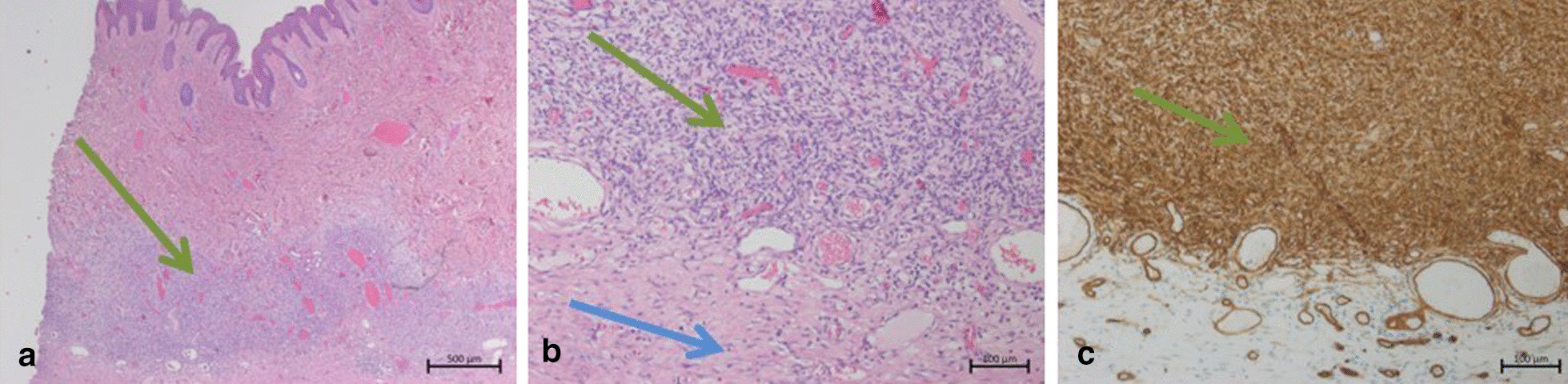
Histopathological image of the dermal DFSP infiltration. Hematoxylin and Eosin (HE) staining shows a dermal infiltration of a basophilic proliferation (green arrow) in accordance with residues of DFSP (**a**). The spindle cell proliferation with minimal atypia, a whorling growth pattern (**b**), and positivity for CD34 (**c**)

Two years before presenting to our outpatient clinic, the patient noticed a skin lesion and subcutaneous swelling on the neck. The lump was neither painful nor tender or noticeable growing. The patient denied any previous trauma or inflammation. After first noticing the skin lesion, an MRI of the neck revealed an encapsulated tumor that was not at high risk of being a malignant process. Therefore, the primary institution did not initiate any therapy at this point. Due to further growth, the patient then underwent primary resection of the tumor without a prior biopsy. The histopathological evaluation revealed malignant skin sarcoma and inadequate safety margins. With the diagnosis of DFSP, the primary institution presented the patient to our interdisciplinary tumor conference for sarcoma. As shown previously for soft tissue sarcomas, revision surgery is mandatory for unexpected sarcoma diagnosis following primary surgery [14]. Two weeks after the first resection, the patient presented at the Department of Plastic and Hand Surgery for secondary resection and subsequent reconstruction. We then initiated an MRI to assess tumor infiltration preoperatively and performed a two-staged procedure with wide resection of the tumor one week after the initial presentation at our outpatient clinic. The extended safety margins needed for the resection are shown in Fig. 1. The resulting defect was closed temporarily with synthetic wound dressing (Epigard^®^). The histopathological examination showed a dermal infiltration of the classical DFSP and postoperative alterations in the deep dermal compartment with uninvolved epidermis and the complete removal of the DFSP with small residues (< 1 cm) of vital tumor cells and satisfying safety margins. In the histological workup, tumor-free margins were > 1 cm to all sides and 0.3 cm to the depth (R-classification: R0). The tumor consists of uniform spindle cells with minimal cytological atypia and woven nuclei arranged in a whorled growth pattern (Fig. 2).

**Fig. 3:**
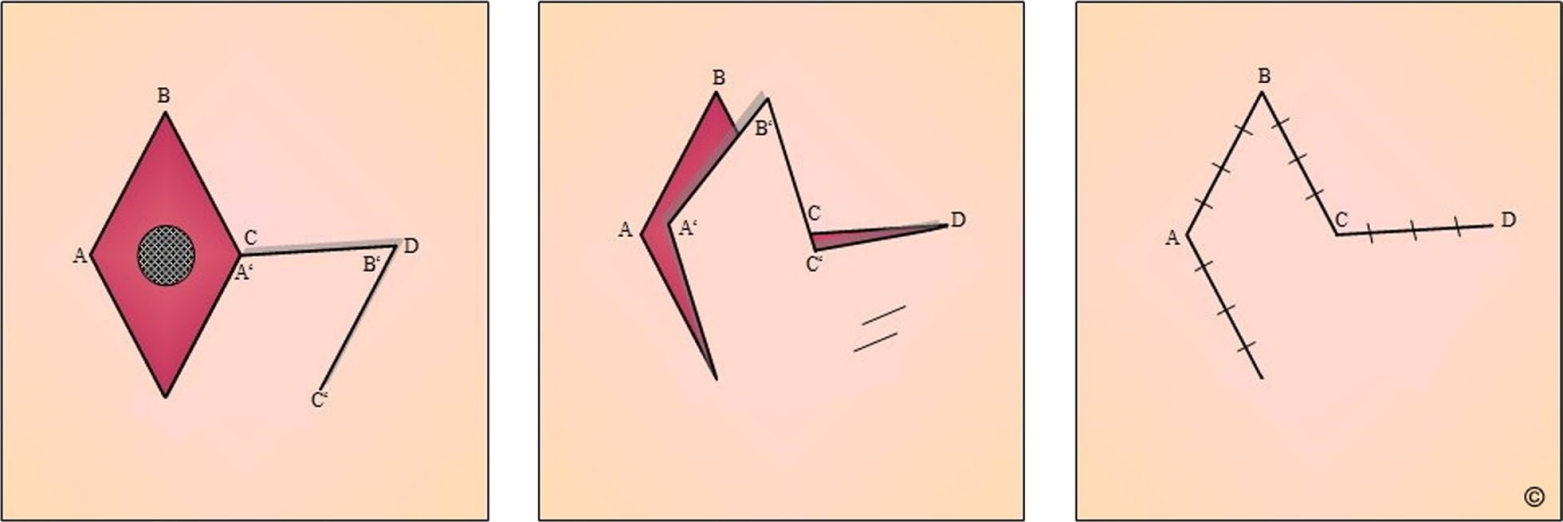
Local flap design for soft tissue coverage. Schematic image of the rhomboid flap (Limberg-Flap) to cover the wound defect

**Fig. 4:**
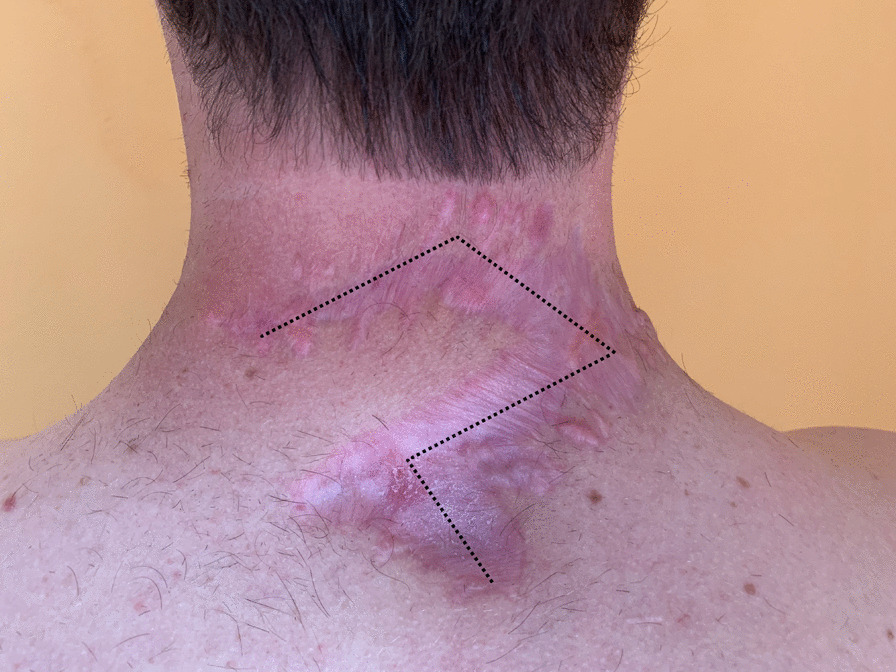
Clinical follow-up 10 months postoperatively. Clinical assessment of the neck with resulting hypertrophic scars ten months postoperatively. The markings indicate the original scar. A prolonged postoperative wound healing and muscle tension led to the visible scar alteration

These safety margins were sufficient considering the initial resection, the complete removal, the small residues of vital tumor cells, and the immunohistochemical workup. Two weeks after the secondary resection, we addressed the remaining wound defect by utilizing a rhomboid skin flap (Limberg-Flap, Fig. 3) [15]. A superficial wound healing disorder prolonged the postoperative course but eventually resolved with non-surgical wound therapy. After discharge, the patient followed up with his general practitioner and dermatologist. Besides oral pain relievers, the patient did not receive any particular medication postoperatively. The localization of the skin tumor and an overall prolonged wound healing resulted in an unsatisfying scar deformity. A secondary revision of the scar, performed by another physician at the patient’s place of residence, led to the visible alteration of the flap geometry (Fig. 4). The recommended after-care included MRI imaging for local recurrence, clinical examination, and regional lymph node ultrasound every 3 months for the first postoperative year. At the last follow-up one year after resecting the DFSP, we did not observe any sign of recurrent tumor growth. Thus, the after-care interval extended to 6 months.

## Discussion and conclusion

DFSP is a rare monoclonal sarcoma arising from the skin and specifically the dermal layers. The tumor initially spreads radially and exhibits vertical growth only at later stages in otherwise healthy patients [16]. Two types of DFSP have been described in the literature. The classical DFSP represents 85% of all tumors and displays a relatively indolent course with low metastatic potential. The remaining 15% of cases exhibit a more aggressive behavior and are therefore described as the fibrosarcomatous high-grade type [17]. As DFSP has been linked to a somatic mutation, it is acquired and non-inherited [18]. However, it is unclear which environmental factors increase the risk of acquiring this genetic deregulation.

Due to the lack of pathognomonic clinical findings and slow growth, DFSP is underrepresented in diagnosis, particularly in patients with devastating skin diseases such as EB. Although DFSP has not yet been described in patients with EB, clinical evidence found in the literature underpins that chronic inflammation might cause DFSP or other skin-related malignancies. DFSP has been reported to arise in areas with a history of prior trauma, including tattoos, surgical scars, burn scars, radiodermatitis, and vaccination sites [19–23]. While the exact mechanism in which EB or tissue trauma may predispose for the development of DFSP is unknown, it is intuitive that long-term stimulation of the immune system at a local level may lead to malignant transformation of dermal cells. The described mechanism and delay to the first occurrence are similar to Marjolin’s ulcer in burn scars [24].

Taken together, the presentation of a DFSP in a young patient with junctional epidermolysis bullosa is likely the result of the chronic inflammation from the underlying skin disease. Nevertheless, this correlation requires further investigation. The overall prognosis of DFSP after adequate excision is generally good. However, a consequent histological workup for every excised skin lesion is mandatory, even if there is no clinical sign for malignancy, especially in patients with chronic skin diseases. An interdisciplinary approach is crucial to ensure clear margins and adequate soft tissue reconstruction.

## Data Availability

Not applicable.

## Abbreviations

EB: Epidermolysis bullosa
SCC: Squamous cell carcinoma
DFSP: Dermatofibrosarcoma protuberans
